# Increase of glandular epithelial cell clusters by an external volume expansion device promotes adipose tissue regeneration by recruiting macrophages

**DOI:** 10.1042/BSR20181776

**Published:** 2019-02-26

**Authors:** Xihang Chen, Yunfan He, Anqi Xu, Zilong Deng, Jingwei Feng, Feng Lu, Yi Yuan

**Affiliations:** 1 Department of Plastic and Reconstructive Surgery, Nanfang Hospital, Southern Medical University, Guangzhou, China; 2 Department of Stomatology, Nanfang Hospital, Southern Medical University, Guangzhou, China; 3 College of Stomatology, Southern Medical University, Guangzhou, China

**Keywords:** adipose tissue regeneration, angiogenesis, external volume expansion, glandular epithelial cell clusters, macrophage

## Abstract

**Background:** There is a clinical need for the use of engineered adipose tissue in place of surgical reconstruction. We previously found that the external volume expansion (EVE) device increased special cell clusters in well-vascularized connective stroma during adipose regeneration. However, the origin of these cell clusters and their role in adipose tissue regeneration remain unknown. **Aim:** In the present study, we evaluated EVE in the construction of expanded prefabricated adipose tissue (EPAT) in a rat model. **Methods:** Rats were randomized into an EVE suction group and a control group, with 24 rats in each group. The structure and origin of the special cell clusters were determined by hematoxylin and eosin staining, and immunohistochemistry; their role in adipose tissue regeneration was investigated by immunohistochemistry and Western blot analyses. **Results:** Special cell clusters began to increase at week 1 with a peak at week 4, and then receded from weeks 8 to 12. Clusters were identified as glandular epithelial cells as determined by their gland-like structure and expression of specific markers. The cell clusters induced significant infiltration of macrophage antigen-2 (Mac-2) positive macrophages by secreting monocyte chemoattractant protein-1 (MCP-1) at the early stage of suction. Subsequently, these infiltrated macrophages expressed massive vascular endothelial growth factor (VEGF) to promoted angiogenesis. **Conclusion:** EVE generated glandular epithelial cell clusters, which recruited macrophages to promote angiogenesis and subsequent adipose tissue regeneration. These findings shed light on the mechanisms underlying the effects of EVE devices on adipose tissue regeneration.

## Introduction

Soft tissue replacement is often required for patients with congenital defects, trauma, or surgical resections [1]. The construction of engineered adipose tissue is a popular research field which addresses the clinical needs resulting from adipose tissue pathologies and defects [2]. External volume expansion (EVE), the external soft-tissue expansion system, has been effectively used in breast plastic surgery [3]. Compared with conventional surgical reconstruction of tissue defects (i.e., transplanting autologous or allogenic tissue), EVE avoided the morbidity in autologous transplants and the immunogenicity of allogenic transplants [4].

The use of EVE devices can result in a significant growth of adipose tissue [5,6]. EVE causes an increase in early inflammatory response and vascular density, creating a local environment in which engineered adipose tissue can potentially benefit [7]. Inflammation is closely related to new adipose tissue formation [8]. Macrophages are key players in the onset of neovascularization and adipogenesis and release angiogenic cytokines, including vascular endothelial growth factor (VEGF) and basic fibroblast growth factor. Recent studies also showed that the infiltration of macrophages precedes endothelial progenitor cells [9], and that common phenotypes are shared between blood-derived macrophages and recruited endothelial progenitor cells, suggesting an even more direct proangiogenic contribution [10]. Studies have found that without macrophages, there was no tissue growth in the engineered adipose tissue [11–13]. Monocyte chemoattractant protein-1 (MCP-1) is best known for its role in recruiting monocytes/macrophages to the arterial wall [14]. And angiogenesis is also closely related to adult neo-adipogenesis [15]. VEGF is one of the key regulators in vascular development [16], which also participates in creating a proper local environment for tissue regeneration. The blockade of VEGF signaling can inhibit *in vivo* adipose tissue formation [15]. However, the causes of local microenvironment changes during the regeneration of adipose tissue with the EVE device warrants further investigation.

Previously, our team developed a rat model using EVE to investigate the mechanism of engineered fat tissue regeneration. Yuan et al. [17] used an EVE device to construct expanded prefabricated adipose tissue (EPAT) in rats, resulting in a significant growth of adipose tissue. They found no significant differences in the structure of fat flaps in the experimental group compared with those of the control group, except for the distribution of the well-vascularized connective tissue. Qin et al. [18] observed that EVE induced the formation of special cell clusters in the well-vascularized connective tissue, which was composed of one or two layers of columnar and/or cuboidal cells, surrounded by a thin layer of slender spindle-shaped cells. The EVE devices even recruited circulating mesenchymal stem cells (MSCs) to participate in adipose tissue regeneration by promoting the secretion of chemokines by columnar and cuboidal cells. Such special cell clusters were also observed in adipose tissue formed in a tissue engineering chamber [19]. However, the origin and the function of these special cell clusters remain unknown.

In the present study, we used an EVE device to construct EPAT in rats and observed the changes of these special cell clusters at specific time points. We investigated the properties of these cells by detecting specific cell markers. In addition, through examining the paracrine functions of these cells, we explored their effects on regulating the changes of the local microenvironment during adipose tissue regeneration.

## Materials and methods

### Animals

All experiments were approved by the Nanfang Hospital Animal Ethics Committee Laboratory and were conducted according to the guidelines of the National Health and Medical Research Council of China. In total, 54 male Sprague–Dawley rats weighing 370 ± 20 g were kept on a 12-h day/night cycle under specific pathogen-free conditions and fed normal chow and water ad libitum.

### External volume expansion model

The EPAT animal model was established as previously described [17]. All rats were briefly anesthetized with isoflurane (30 mg/kg body weight), and their inguinal fat flaps were exposed with an incision in the unilateral inguinal area to the right side. The fat flaps were separated from the surrounding tissue and transferred to the middle of the abdomen. Finally, the incisions were sutured, and the contours of the flaps were marked on the skin.

**Figure 1:**
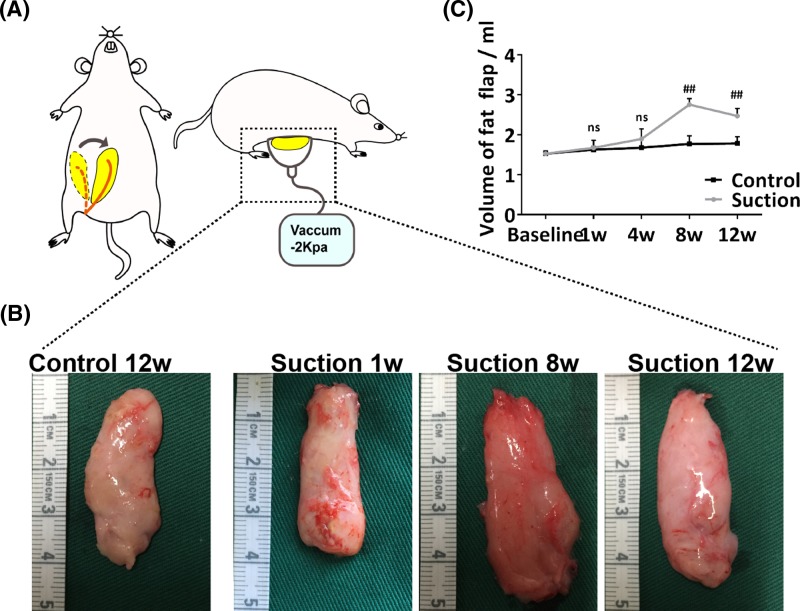
Regeneration of adipose tissue by using an EVE device (**A**) Illustration of our previously reported EVE model. (**B**) Macroscopic views of harvested adipose tissue of baseline and suction group at weeks 1, 8 and 12 (**C**) Adipose tissue volumes. The results showed that sample volume increased from baseline to week 8 in the EVE group. Although the volume declined slightly after EVE device removal, the volumes of the adipose tissues at week 12 were larger than that of the control group. (Student’s *t*-test, ^##^*P*<0.01 compared with the control group).

Six rats were used to measure the baseline volume of fat flaps. The other 48 rats were randomized into two groups of 24, an EVE group and a control group. Suction was applied in the EVE group with a dome-shaped acrylonitrile-styrene copolymer device, with a diameter of 2 cm and internal volume of 10 ml, connected to a suction pump (low-vacuum suction unit DYX-1A, SMAF, Shanghai, P.R. China) via a flexible rubber hose at a constant pressure of 2 kpa. Suction was applied to the transferred flap after surgical recovery for 6 to 8 h per day for eight consecutive weeks. After 8 weeks, suction was discontinued, and the rats were observed for an additional 4 weeks. The fat flaps received no suction in the control group (Figure 1A).

### Tissue collection

Animals were killed at weeks 1, 4, 8, and 12 (*n*=6 for each time point), and adipose tissue was separated from the surrounding tissues and harvested. Its volume was measured based on the volume of fluid displacement. The tissue samples were cut in half, vertically through the longitudinal axis; one half was stored at −80°C for protein analysis, and the other half was fixed in 4% paraformaldehyde for 24 h and embedded in paraffin for histologic evaluation.

### Histologic examination

The paraffin-embedded samples were cut into 3 μm thick sections, which were stained with hematoxylin and eosin for conventional morphologic evaluation. All samples were assessed with an Olympus DP71 digital camera (Olympus Corp., Tokyo, Japan). The ratio of the area of special cell clusters to the area of the whole slice section was determined using Image J software. Three slices of every sample were measured.

### Immunohistochemistry of special cell clusters specific markers

To investigate the origin of these special cell clusters, specific cell markers were detected by using immunohistochemistry, following the manufacturer’s standard procedure as below. Paraffin sections were dewaxed and hydrated before immunohistochemical staining. Slides were washed with PBS and heat remediated with citrate buffer. They were then incubated overnight with the following primary antibodies: rabbit anti-rat Cytokeratin 8 antibody (ab32579, 1:500; Abcam, Cambridge, U.K.), Cytokeratin 18 antibody (ab133263, 1:250; Abcam), Cytokeratin 5 antibody (ab52635, 1:250; Abcam), E-cadherin antibody (ab40772, 1:500; Abcam), anti-α smooth muscle Actin (α-SMA) (ab21027, 1:250; Abcam); followed by the secondary antibody, horseradish peroxidase(HRP)-conjugated goat anti-rabbit immunoglobulin G(IgG) heavy and light chains (H&L) (ab6721,1:1000; Abcam) for 30 min. Next, 3,3′-Diaminobenzidine chromogen was applied to the slides for histological staining. All samples were assessed with an Olympus DP71 digital camera (Olympus Corp., Tokyo, Japan).

### Immunohistochemistry and Immunofluorescence of specific role of these special cell clusters

To investigate the specific role of these special cell clusters, the following primary antibodies were applied according to the standard procedure of immunohistochemistry described above: rabbit anti-rat CD31 antibody (ab182981, 1:2000; Abcam), MCP-1 antibody (A7277, 1:100; ABclonal), macrophage antigen-2 (Mac-2) (ab76245, 1:250; Abcam). Angiogenesis was analyzed by measuring the blood vessel (CD31-positive cells) density (vessels/mm^2^) in the whole field (400×). Three slices were taken from each sample and five visual fields (400×) containing the special cell clusters were randomly selected in each slice. Macrophages were assessed by counting the Mac-2 positive cells in the visual fields containing the special cell clusters.

The secretion of MCP-1 by these special cell clusters and the secrection of VEGF by macrophages were detected by immunofluorescent technique. Paraffin sections were incubated overnight with the following primary antibodies: rabbit anti-rat MCP-1 antibody (ab7202, 1:100; Abcam), Mac-2 antibody (ab76245, 1:100, Abcam) and mouse anti-rat Pan-CK antibody (ab215838, 1:200; Abcam); VEGF antibody (ab1316, 1:100; Abcam), and then followed by Alexa Fluor 594-conjugated goat anti-rabbit immunoglobulin G (ab150080, 1:1000; Abcam) and Alexa Fluor 488-conjugated goat anti-mouse immunoglobulin G (ab150113, 1:1000; Abcam). Nuclei were stained with 4′,6-diamidino-2-phenylindole (D8417, Sigma Aldrich). The images were obtained using a confocal microscope system (Carl Zeiss, Jena, Germany).

### Western blot analysis

Total cell lysates from the adipose samples were prepared using a fat tissue protein extraction kit (BB-312262; BestBio, Nanjin, China). The protein concentration was estimated using the BCA protein assay (Thermo-Fisher Scientific, Waltham, MA). Protein extracts were subjected to sodium dodecyl sulfate polyacrylamide gel electrophoresis using the NuPage electrophoresis system and then transferred to Immobilon polyvinylidene difluoride membranes (Millipore, Billerica, Mass.). Membranes were blocked in 5%- albumin from bovine serum and immunoblotted with primary antibodies: rabbit anti-rat Mac-2 antibody (ab76245, 1:5000; Abcam), VEGF antibody (A0280, 1:1000; ABclonal), and MCP-1 antibody (A7277, 1:1000; ABclonal). After incubation with secondary antibodies, the detection was performed with the Western Breeze Chemiluminescent Detection Kit (ThermoFisher Scientific). Glyceraldehyde-3-phosphate dehydrogenase (GAPDH) was used as an internal control.

### Statistical analysis

Data were expressed as means ± S.E.M. Independent Student’s *t*-tests were used to compare two groups at a single time point. The difference among four time points in the suction group was tested by one-way analysis of variance (ANOVA) followed by Bonferroni’s *post hoc* analysis or Tamhane’s T2 *post hoc* test. A value of *P*<0.05 was considered statistically significant.

## Results

### Volume changes of engineered adipose tissue

The volume of the fat flaps in the suction group increased from week 1 (1.67 ± 0.19 ml) to week 8 (2.76 ± 0.15 ml). A slight decrease of the volume of fat flaps occurred after EVE removal, but the volume was still larger than that of control group. The volume did not change significantly over time in the control group. (*P*<0.01) (Figure 1B,C).

### The number of special cell clusters peaked at week 4 then gradually receded

**Figure 2:**
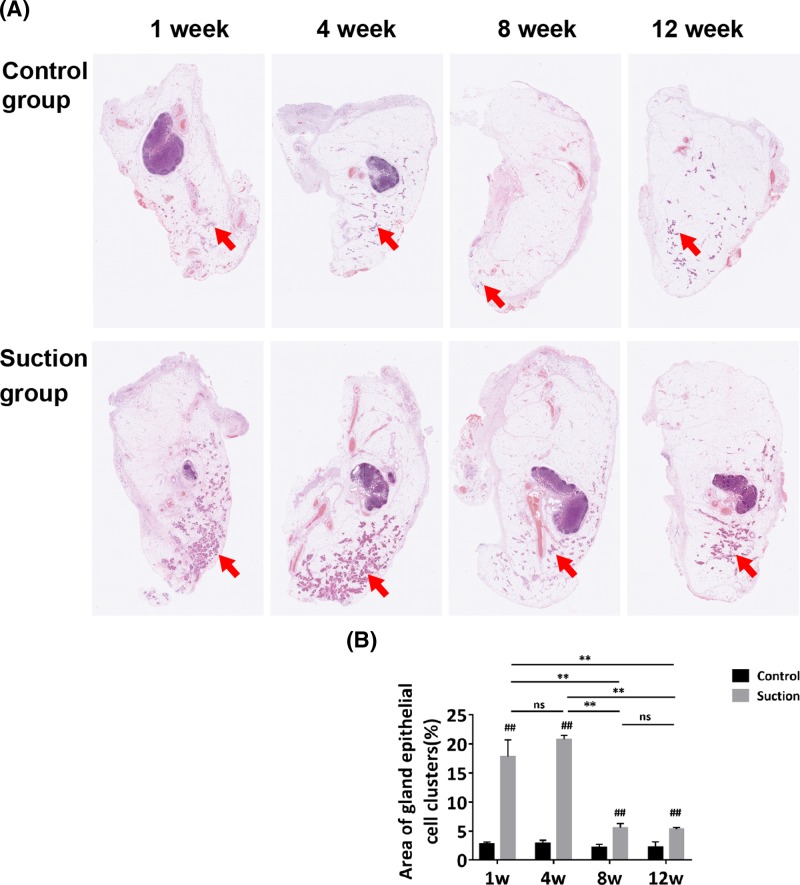
Histologic analysis of samples by hematoxylin and eosin (**A**) Harvested samples were subjected to hematoxylin and eosin staining.(Magnification = 20×) (**B**) Special cell clusters cells (red arrows) grew at week 1 and peaked at week 4 then decreased at weeks 8 and 12. But were still larger than that of control group at each time point. Red arrows indicated the special cell clusters. (Student’s *t*-est, ^##^*P*<0.01 compared with the control group; one-way ANOVA followed by Bonferroni’s *post hoc* analysis, ^**^P<0.01; ns represents not significant).

There were no significant differences in the structure of fat flaps in the suction group compared with the control group, except for the distribution of the special cell clusters (Figure 2A). The ratio of special cell clusters started to increase at week 1 (17.87 ± 2.85%), reached a peak at week 4 (20.81 ± 0.67%), and gradually decreased at week 8 (5.62 ± 0.72%) and week 12 (5.39 ± 0.28%) (*P*<0.01). The ratios in the suction group were higher than those of the control group at each time point (*P*<0.01) (Figure 2B).

### Special cell clusters were identified as glandular epithelial cell clusters

**Figure 3:**
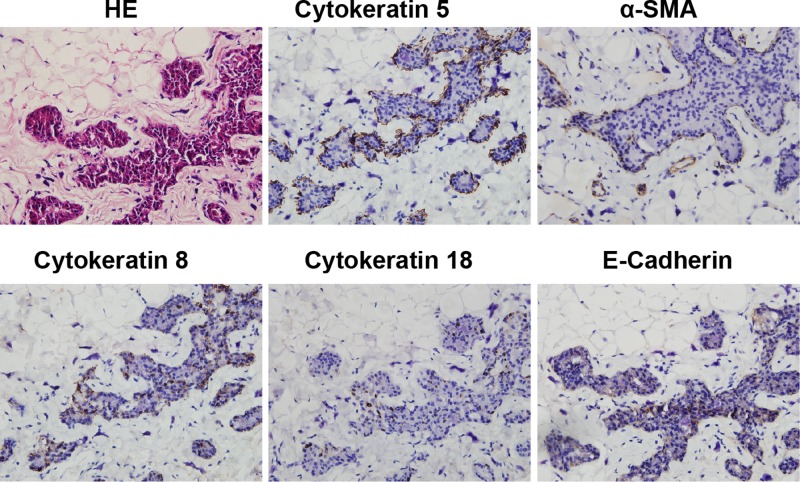
Immunohistochemistry of specific markers in special cell clusters Immunohistochemistry was performed to detect the expression of Cytokeratin 5, α-SMA, Cytokeratin 8, Cytokeratin 18 and E-cadherin in samples. (Magnification = 400×).

Special cell clusters were composed of inner layers of columnar-shaped cells and outer layers of spindle-shaped cells, manifesting a lumen-like structure in morphology (Figure 3). We determined the origin of cells by examining their specific markers. The spindle-shaped cells expressed Cytokeratin 5 and α-SMA (Figure 3), while the columnar-shaped cells expressed Cytokeratin 8 and Cytokeratin 18; in addition, these cells may interact with each other through E-cadherin (Figure 3). These results suggested that the special cell clusters were glandular epithelial cell clusters, composed of spindle-shaped myoepithelial cells and columnar-shaped luminal epithelium cells.

### Glandular epithelial cells recruited macrophages by secreting MCP-1

**Figure 4:**
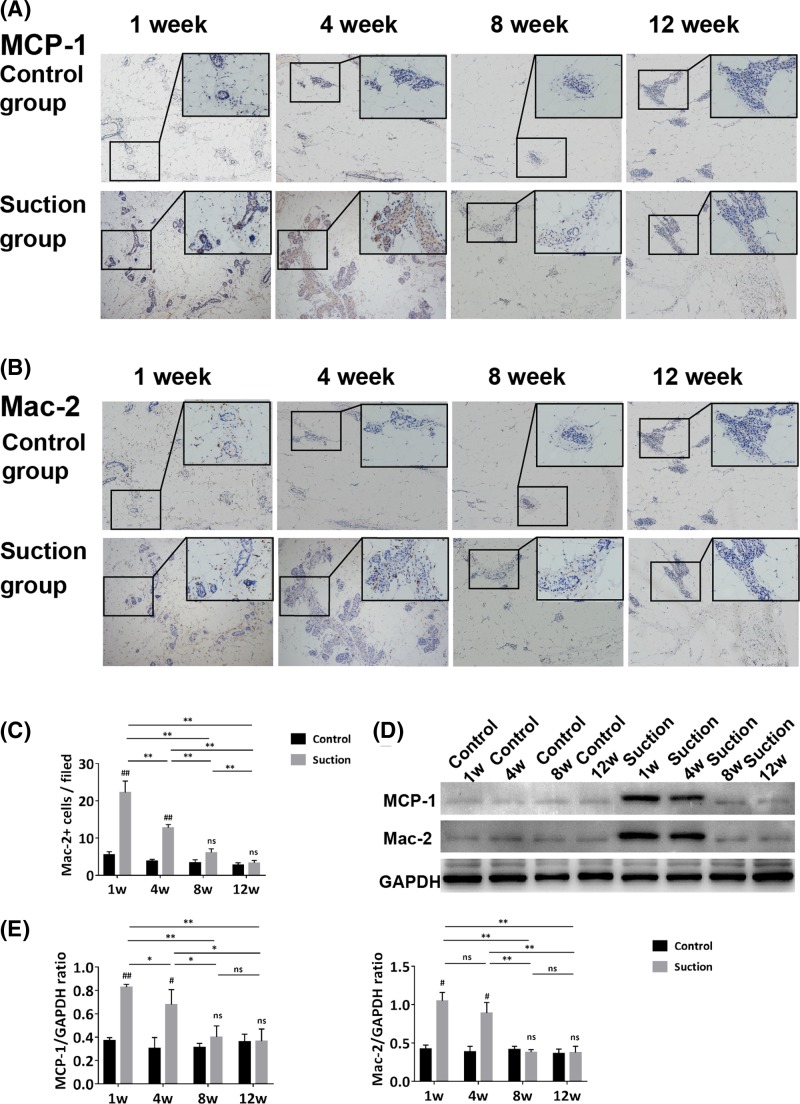
Specific role of glandular epithelial cells as detected by Immunohistochemistry (**A**) Immunohistochemistry of MCP-1 in both groups at each time point. MCP-1 was strongly expressed in glandular epithelial cells at week 1 and 4, while slightly expressed at week 8 and 12, and only occasionally observed in the control group. (**B**) Immunohistochemistry of Mac-2 in both groups at each time points. Mac-2 was greatly up-regulated at week 1 and 4, then decreased over time, and was only sparsely distributed in the control group. (**C**) Statistical analysis of Mac-2-positive cells was performed among the four time points in both groups. (**D**) The Western blot of MCP-1 and Mac-2 at the four time points in both groups. (**E**) Statistical analysis of MCP-1 and Mac-2 expression of Western blot in both groups at each time points. (Magnification = 100×; Student’s *t*-test, ^#^*P*<0.05, ^##^*P*<0.01 compared with the control group; one-way ANOVA followed by Bonferroni’s *post hoc* test or Tamhane’s T2 *post hoc* test analysis, *P<0.05, **P<0.01; ns represents not significant).

The immunohistochemistry analysis in the suction group demonstrated that MCP-1 was strongly expressed in glandular epithelial cells at week 1 and 4, and was slightly expressed at week 8 and 12, which showed no obvious difference to the expression in the control group (Figure 4A). The co-expression of MCP-1 and Pan-CK (the specific marker of glandular epithelial cells) was also confirmed by immunofluorescence. In suction group, many MCP-1+/Pan-CK+ cells were detected at week 1 and 4, with fewer detected over time (See additional figure 1). Likewise, Western blot analysis showed that MCP-1 expression in the suction group was higher than those of the control group at week 1 and 4 (*P*<0.01), and the expression of MCP-1 in the suction group peaked at week 1, and gradually decreased during the following weeks (*P*<0.05) (Figure 4D,E).

The immunohistochemistry analysis of Mac-2 was conducted to detect macrophages. As shown in Figure 4B, the samples of the suction group at week 1 and 4 contained a large number of macrophages, which were mainly distributed in the extracellular matrix around or within the glands. Quantification of Mac-2-positive cells indicated that macrophages in the suction group peaked at week 1 (22.56 ± 3.03/field), then decreased at later time points (*P*<0.01). Although the number of macrophages in the suction group was higher than that in control group at the first three time points, the difference disappeared at week 12 (*P*<0.01) (Figure 4C). Western blot analysis also confirmed these results (Figure 4D,E).

### Macrophages promoted angiogenesis by secreting VEGF at the early stage

**Figure 5:**
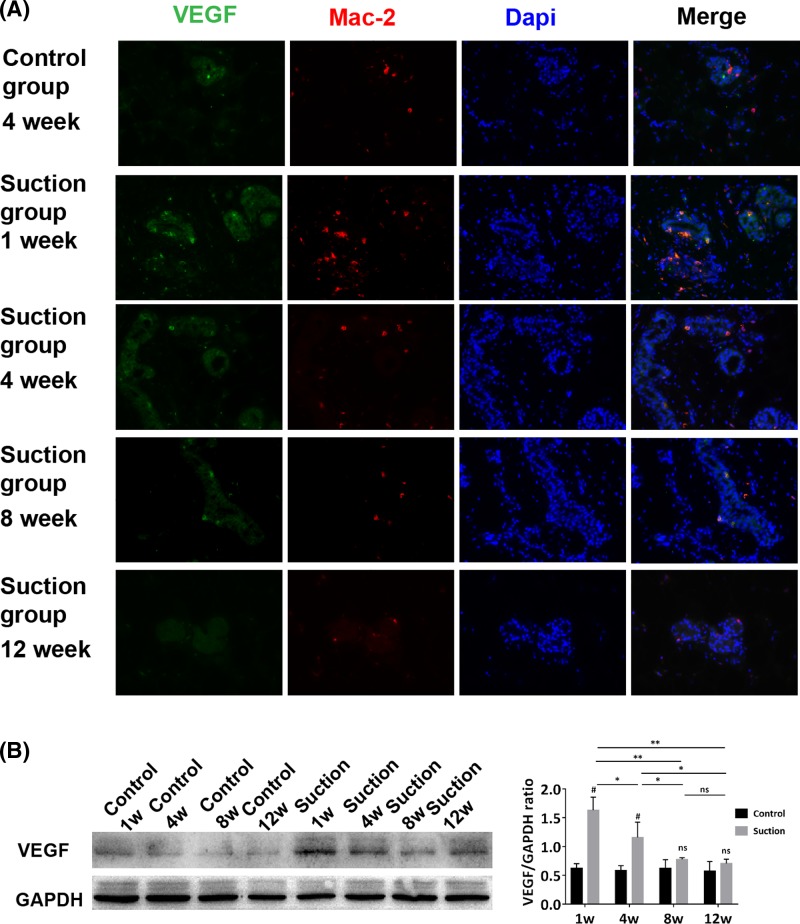
Mac-2 and VEGF were stained in sections of samples at different time points (**A**) Large number of Mac-2+/VEGF+ cells could be detected surrounding glandular epithelial cell clusters at week 1 in the suction group, but their number decreased over time. They were only occasionally observed in the control group. (**B**) Western blot analysis of expression of VEGF in both groups. (Magnification = 400×; Student’s *t*-test, ^#^*P*<0.05 compared with the control group; one-way ANOVA followed by Bonferroni’s *post hoc* analysis, ^*^P<0.05, ^**^P<0.01; ns represents not significant).

Sections obtained from samples at each time point were examined using Mac-2 and VEGF antibodies. In the suction group, many Mac-2+/VEGF+ cells were detected surrounding glandular epithelial cell clusters at week 1, with fewer detected over time. However, there were fewer Mac-2+/VEGF+ cells in the control group than in the suction group (Figure 5A). VEGF were also detected by Western blotting. VEGF was up-regulated at week 1 and then decreased over time in suction group (*P*<0.05). The VEGF expression level in the control group was lower than that in the suction group at week 1 and 4 (*P*<0.05) (Figure 5B).

### The increase of glandular epithelial cell clusters accompanied with angiogenesis

**Figure 6:**
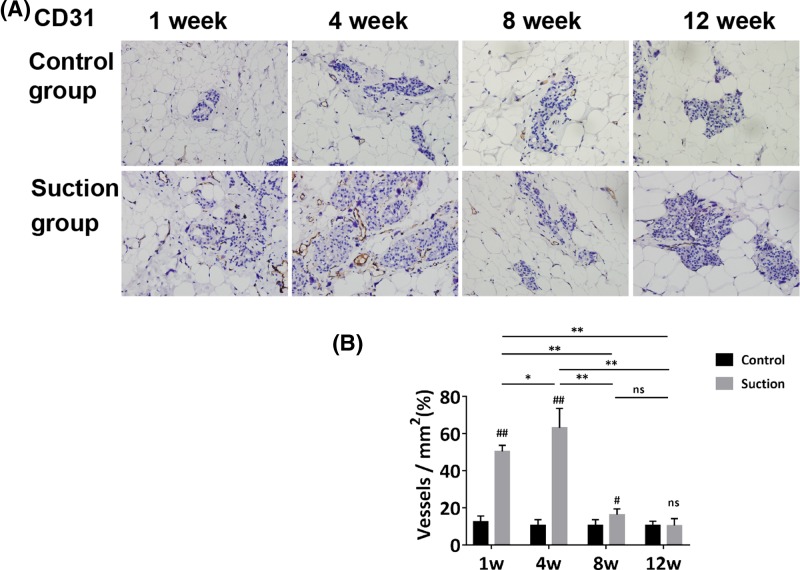
The changes of new blood vessel in adipose tissue samples observed by Immunohistochemistry (**A**) CD31-positive cells were considered as the new blood vessels in the samples. New blood vessels were mainly distributed in the extracellular matrix around the glands. Their density was increased at week 1 and 4, then decreased over time in the suction group. In contrast, they were only occasionally observed in the control group at each time point. (**B**) Statistical analysis was performed among the four time points. Although the blood vessels density of the suction group was higher than that of control group at weeks 1, 4, and 8, the difference disappeared at week 12. (Magnification = 400×; Student’s *t-*test, ^#^*P*<0.05, ^##^*P*<0.01 compared with the control group; one-way ANOVA followed by Tamhane’s T2 *post hoc* test analysis, ^*^P<0.05, ^**^P<0.01; ns represents not significant).

The immunohistochemistry analysis demonstrated that the samples in the suction group at week 1 and 4 contained many newly formed blood vessels. By observing the distribution of angiogenesis (CD31-positive cells), we found that new blood vessels were mainly distributed in the extracellular matrix around the glands (Figure 6A). Quantification analyses showed that the blood vessel density of the suction group increased from week 1 (50.36 ± 3.35 vessels/mm^2^) to week 4 (63.13 ± 10.48 vessels/mm^2^), then dramatically decreased at later time points (week 8: 16.21 ± 3.12 vessels/mm^2^ and week 12: 10.42 ± 3.74 vessels/mm^2^) (*P*<0.05). Although the blood vessel density of the suction group was higher than that of the control group at weeks 1, 4, and 8, the difference disappeared at week 12 (*P*<0.05) (Figure 6B).

## Discussion

In the present study, we showed that the EVE device induced significant adipose tissue regeneration as previously described [17], and found the number of special cell clusters increased from weeks 1 to 4, but decreased at week 8 and 12 with use of the EVE device. These changes implicated the potential role of special cell clusters in regulating fat tissue regeneration. However, it was necessary for us to identify the exact origin of these specific cell clusters before exploring their specific role.

We found that these special cell clusters were composed of inner layers of columnar-shaped cells and outer layers of spindle-shaped cells, which morphologically resembled the structure of glands [20,21]. Therefore, we examined the expression profile of gland-related specific markers in the special cell clusters. As expected, the columnar cells expressed Cytokeratin 8, Cytokeratin 18, and E-cadherin, which indicated that these cells were a type of luminal epithelium cell [22–24]; the spindle-shaped cells expressed Cytokeratin 5 and α-SMA, which suggested that they were myoepithelial cells [25]. Because myoepithelial cells and lumen epithelial cells are usually the two types of cells that make up the glandular structure tissue [26,27], these results showed that the increasing ‘special cell clusters’ under the action of EVE are glandular epithelial cell clusters.

Epithelium cells are mechanically sensitive cells, which are inclined to proliferate under the action of external forces [28]. Therefore, we speculated that the external force exerted by the EVE device resulted in the proliferation of the glandular epithelial cell clusters at the early stage during adipose tissue regeneration. However, as Wyatt et al. [29] summarized in their review, ‘the majority of any imposed stress is likely to be rapidly dissipated by cytoskeleton turnover and membrane dynamics in cells, even if some stress may remain at long time scales’. Therefore, gland regression at the later stage in the suction group may be attributed to the adaptability of tissues or cells to the external microenvironment.

We also found that angiogenesis appeared around glandular epithelial cell clusters. The number of newly formed blood vessels peaked at week 4 and gradually declined during the following weeks. There were close spatial and temporal inter-relationships between blood vessel formation and adipogenesis, and the sprouting of new blood vessels from preexisting vasculature was coupled to adipocyte differentiation [30]. In the early stages of adipose regeneration, angiogenesis is fundamental for the effective development or regeneration of adipose tissue and is a hallmark of an established reconstruction response [31,32]. Considering that a similar trend was observed for glandular epithelial cell clusters during the adipose regeneration process, we questioned whether the increase of glandular epithelial cell clusters is involved in angiogenesis.

To explore the mechanisms by which glandular epithelial cells promoted angiogenesis, we focused on their paracrine effect. We found that MCP-1 was strongly expressed in glandular epithelial cells in the suction group at week 1 and 4, at which time macrophages were also present within or around the glandular epithelial cells. MCP-1 can be produced by various cell types, including epithelial cells, endothelial cells, smooth muscle cells, fibroblasts, monocytes, and adipocytes [33]. The breast epithelium cells normally express a small amount of MCP-1, but the expression of MCP-1 will increase under special situations (e.g., involution) [34]. MCP-1 has been proposed to play critical roles in recruitment of monocytes into adipose tissue. When MCP-1 was deleted in mice, the number of macrophages found in adipose tissue was significantly reduced [35,36], and when MCP-1 was up-regulated in mice, more macrophages were founded in adipose tissue [37]. And recruitment of macrophages to obese adipose tissues can occur through the engagement of CCR2, the receptor for MCP-1, which is expressed on peripheral monocytes/macrophages [38]. In our study, we found that the expression of MCP-1 and Mac-2 were both elevated at week 1 and 4 in suction group. Considering the regulatory effect of MCP-1 on macrophages infiltration, we speculated that mechanical force may have stimulated the increased expression of MCP-1 in glandular epithelial cells, and subsequently, the enrichment of macrophages.

A role for macrophages is assumed in the onset of angiogenesis, and the regulation of adipogenesis. Nishimura et al. reported that lectin-binding cell clusters, which are positive for both CD68 and F4/80, belong to the monocyte/macrophage lineage and might constitute adipogenic/angiogenic cell clusters [30]. Macrophages can stimulate angiogenesis in a VEGF-dependent manner involving RhoA/Rho kinase signaling, which would also induce endothelial cell migration [39]. VEGF-expressing macrophages have been reported to induce the vascular sprout and tissue growth during the early stage of the repair response [40]. Similarly, the macrophages represent the major proportion of inflammatory cells in the fat graft environment and are closely related to vessel formation and are important in blood-derived stem cell recruitment at the early stage of fat grafting [12]. In our study, we found that the recruited macrophages expressed massive amounts of VEGF at week 1 in suction group, and the paracrine effect of macrophage may promote angiogenesis during fat regeneration under EVE device.

## Conclusion

In summary, we found that the increased special cell clusters generated by EVE were identified as glandular epithelial cell clusters. We believe our study demonstrated that the increase of glandular epithelial cell clusters exerted by EVE promoted angiogenesis by recruiting macrophages during adipose tissue regeneration. These findings shed light on the mechanisms underlying the effects of EVE devices on adipose tissue regeneration.

## Supporting information

**Supplementary Figure:** 

## Abbreviations

ANOVA: analysis of variance
EPAT: expanded prefabricated adipose tissue
EVE: external volume expansion
H&L: heavy and light chains
HRP: horseradish peroxidase
IgG: immunoglobulin G
Mac-2: macrophage antigen-2
MCP-1: monocyte chemoattractant protein-1
VEGF: vascular endothelial growth factor
