# Supplementary figures and images for: Increase of glandular epithelial cell clusters by an external volume expansion device promotes adipose tissue regeneration by recruiting macrophages
Source: Biosci Rep. 2019 Feb 26;39(2):BSR20181776. doi: 10.1042/BSR20181776 (PMC6390125; doi:10.1042/BSR20181776)

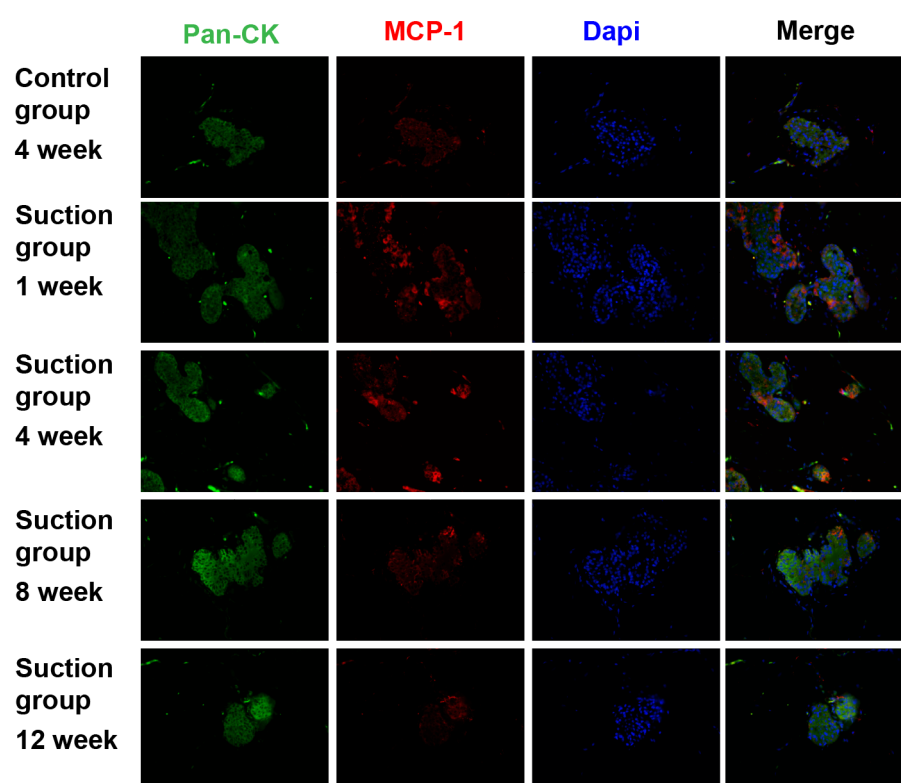

Supplement: Supplementary file 1 [file bsr-39-bsr20181776_Supp1.pdf]
